# Supplementary material for: Hybrid Models and Biological Model Reduction with PyDSTool
Source: PLoS Comput Biol. 2012 Aug 9;8(8):e1002628. doi: 10.1371/journal.pcbi.1002628 (PMC3415397; doi:10.1371/journal.pcbi.1002628)
Supplement: Text S4 — Complete source code for the PyDSTool package (version 0.88.120504). Includes API documentation and help files linking to web pages. This file is identical to the current public release on Sourceforge.net. (ZIP) [file pcbi.1002628.s004.zip › PyDSTool/html/toc-PyDSTool.Toolbox.optimizers.criterion-module.html]

xml version="1.0" encoding="ascii"?


criterion


# Module criterion

---

## Classes

AICCriterion  
 AbsoluteParametersCriterion  
 AbsoluteValueCriterion  
 AndComposition  
 GradientCriterion  
 IterationCriterion  
 ModifiedAICCriterion  
 MonotonyCriterion  
 OrComposition  
 RelativeParametersCriterion  
 RelativeValueCriterion  

## Functions

criterion  

## Variables

criterion\_\_all\_\_

---

[hide private]
